# (3*R*,4*R*,5*S*)-4-Hydr­oxy-3-methyl-5-[(2*S*,3*R*)-3-methyl­pent-4-en-2-yl]-4,5-dihydro­furan-2(3*H*)-one

**DOI:** 10.1107/S1600536809050399

**Published:** 2009-11-28

**Authors:** Annika Gille, Markus Schürmann, Hans Preut, Martin Hiersemann

**Affiliations:** a Fakultät Chemie, Technische Universität Dortmund, Otto-Hahn-Strasse 6, 44221 Dortmund, Germany

## Abstract

The relative configuration of the title compound, C_11_H_18_O_3_, which was synthesized using a catalytic asymmetric Gosteli–Claisen rearrangement, a diastereoselective reduction with K-Selectride and an Evans aldol addition, was corroborated by single-crystal X-ray diffraction analysis. The five-membered ring has an envelope conformation with a dihedral angle of 29.46 (16)° between the coplanar part and the flap (the hydr­oxy-bearing ring C atom). In the crystal, mol­ecules are connected *via* bifurcated O—H⋯(O,O) hydrogen bonds, generating [010] chains.

## Related literature

For further synthetic details, see: Abraham *et al.* (2001 ▶, 2004 ▶); Brown (1973 ▶); Evans *et al.* (1981 ▶, 1999 ▶); Otera *et al.* (1992 ▶). For the structure of the major diastereisomer arising from the same reaction, see: Gille *et al.* (2008 ▶).
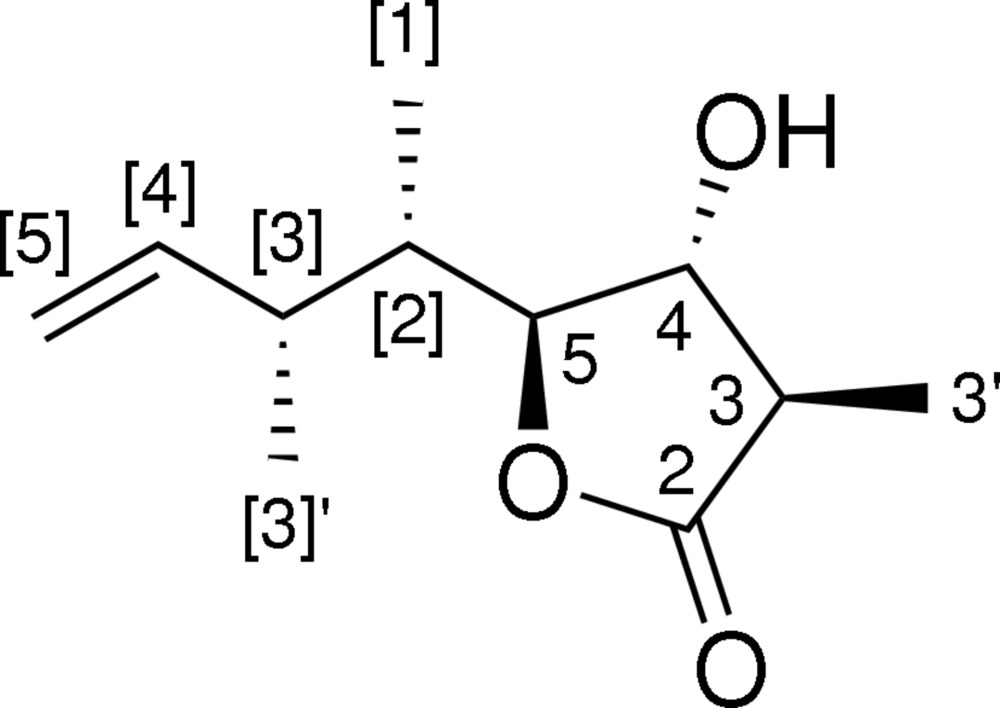


## Experimental

### 

#### Crystal data

C_11_H_18_O_3_
*M* *_r_* = 198.25
Monoclinic, 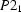

*a* = 7.7265 (10) Å
*b* = 6.4798 (8) Å
*c* = 11.0598 (16) Å
β = 92.563 (14)°
*V* = 553.17 (13) Å^3^
*Z* = 2
Mo *K*α radiation
μ = 0.09 mm^−1^
*T* = 173 K
0.50 × 0.18 × 0.04 mm

#### Data collection

Oxford Diffraction Xcalibur S CCD diffractometer
Absorption correction: none
3255 measured reflections
1129 independent reflections
737 reflections with *I* > 2σ(*I*)
*R*_int_ = 0.040

#### Refinement

*R*[*F* ^2^ > 2σ(*F* ^2^)] = 0.035
*wR*(*F* ^2^) = 0.048
*S* = 0.99
1129 reflections
131 parameters
1 restraint
H-atom parameters constrained
Δρ_max_ = 0.14 e Å^−3^
Δρ_min_ = −0.15 e Å^−3^

### 

Data collection: *CrysAlis CCD* (Oxford Diffraction, 2008 ▶); cell refinement: *CrysAlis CCD*; data reduction: *CrysAlis CCD*; program(s) used to solve structure: *SHELXS97* (Sheldrick, 2008 ▶); program(s) used to refine structure: *SHELXL97* (Sheldrick, 2008 ▶); molecular graphics: *SHELXTL-Plus* (Sheldrick, 2008 ▶); software used to prepare material for publication: *SHELXL97* and *PLATON* (Spek, 2009 ▶).

## Supplementary Material

Crystal structure: contains datablocks I, global. DOI: 10.1107/S1600536809050399/hb5238sup1.cif

Structure factors: contains datablocks I. DOI: 10.1107/S1600536809050399/hb5238Isup2.hkl

Additional supplementary materials:  crystallographic information; 3D view; checkCIF report

## Figures and Tables

**Table 1:** Hydrogen-bond geometry (Å, °)

*D*—H⋯*A*	*D*—H	H⋯*A*	*D*⋯*A*	*D*—H⋯*A*
O3—H3⋯O1^i^	0.84	2.52	3.023 (2)	120
O3—H3⋯O2^i^	0.84	2.10	2.931 (3)	171

Symmetry code: (i) 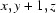.

**Fig. 1.**
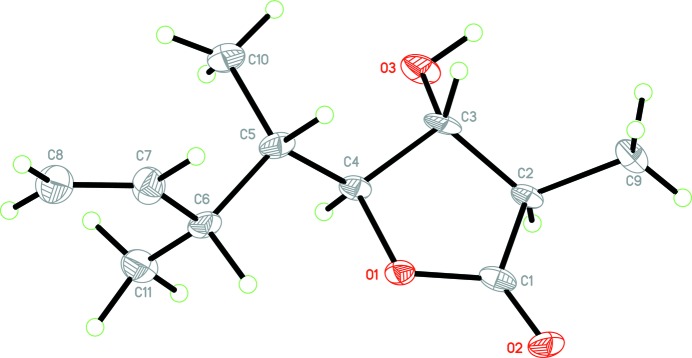
: The molecular structure of (I) with displacement ellipsoids shown at the 30% probability level.

## supplementary crystallographic information

### Comment

The title compound, (I), was synthesized using a catalytic asymmetric Gosteli-Claisen rearrangement (Abraham *et al.*, 2001; Abraham *et al.*, 2004), a diastereoselective reduction with K-Selectride (Brown, 1973) and an Evans aldol addition (Evans *et al.*, 1981). In order to verify the relative configuration of the obtained diastereomeric aldol adducts, 4-(*tert*-butyldimethylsilyloxy)-3-hydroxy-2,5,6-trimethyloct-7-enoyl) -4-isopropyloxazolidin-2-one, (III), the γ-lactones (II) and (I) were prepared by removal of the silyl protecting group (Otera *et al.*, 1992) and subsequent *in situ *lactonization. The diastereomeric mixture of the γ-lactones could be separated by column chromatography. An X-ray crystal structure analysis of the major diastereomer (II) has already been published (Gille *et al.*, 2008). Fig. 1 depicts the structure of the isolated minor diastereomer (I). The configuration of the chiral C atoms in (I) can be attributed to the stereochemical course of the Evans aldol addition (C3 *R* and C4 *R*), the diastereoselective reduction with K-Selectride (C5 *S*) and the catalytic asymmetric Claisen rearrangement (C[2] *S* and C[3] *R*) using the chiral Lewis acid [Cu{(*S,S*)-*tert*-butyl-box}](H_2_O)_2_(SbF_6_)_2_ (Evans *et al.*, 1999).

### Experimental

The title compound, (I), was synthesized from the corresponding aldol adduct, (III), using tetrabutylammonium fluoride (TBAF) in the presence of acetic acid (Otera *et al.*, 1992) to remove the silyl protecting group. The subsequent lactonization proceeded *in situ *.

To an ice-cooled solution of crude (III) (dr = 49/51, 0.04 g, 0.10 mmol, 1 eq) in THF (1 ml, 11 ml/mmol III) was added a solution of AcOH (0.5 µl, 0.010 mmol, 0.1 eq) in THF (0.1 ml, 1.1 ml/mmol III) and TBAF (1 *M* in THF, 0.11 ml, 0.11 mmol, 1.1 eq). After 15 min at 273 K, the reaction mixture was diluted by the addition of saturated aqueous NH_4_Cl solution. The aqueous layer was extracted with CH_2_Cl_2_ (4x) and the combined organic phases were dried (MgSO_4_) and concentrated under reduced pressure. Purification by flash chromatography (crude product charged on silica gel, cyclohexane/ethyl acetate 10/1 to 5/1) afforded lactone (I) (0.006 g, 0.03 mmol, 30%) as a single diastereomer and additionally a mixture of (I) and the diastereomer (II) (0.013 g, 0.07 mmol, 69%, dr = 70/30) as colourless crystals. Subsequent recrystallization of (I) by vapor diffusion technique from isohexane and ethyl acetate provided a colourless plate of (I) single-crystal suitable for an X-ray crystal structure analysis. *R_f_* 0.35 (cyclohexane/ethyl acetate 2/1); mp 378 K; ^1^H NMR (CDCl_3_, 400 MHz, δ): 0.92 (d, ^3^*J* = 7.1 Hz, 3H), 1.00 (d, ^3^*J* = 7.0 Hz, 3H), 1.32 (d, ^3^*J* = 7.2 Hz, 3H), 1.85 (dqd, ^3^*J* = 8.2, 7.1, 4.9 Hz, 1H), 2.1 (br. s, 1H), 2.51–2.63 (m, 1H), 2.63 (dq, ^3^*J* = 8.4, 7.2 Hz, 1H), 3.92 (dd, ^3^*J* = 7.4, 8.2 Hz, 1H), 4.07 (dd, ^3^*J* = 8.4, 7.4 Hz, 1H), 5.04 (dd, ^3^*J(E)* = 17.8 Hz, ^2^*J* = 1.3 Hz, 1H), 5.05 (dd, ^3^*J(Z)* = 10.0 Hz, ^2^*J* = 1.3 Hz, 1H), 5.81 (ddd, ^3^*J(E)* = 17.8 Hz, ^3^*J(Z)* = 10.0 Hz, ^3^*J* = 6.8 Hz, 1H); ^13^C NMR (CDCl_3_, 101 MHz, δ): 10.1 (CH_3_), 12.9 (CH_3_), 13.7 (CH_3_), 37.8 (CH), 41.4 (CH), 44.4 (CH), 78.0 (CH), 84.7 (CH), 114.5 (CH_2_), 142.6 (CH), 176.4 (C); IR (cm^-1^): 3400(br,s) (ν O—H, OH in H-bridges), 3085(w) (ν C—H, olefin), 2970(*s*) 2925(*s*) 2890(*m*) 2855(*m*) (ν C—H, CH, CH_3_), 1735(*s*) (ν C=O, lactone), 1640(w) (ν C=C), 1455(*m*) (δ_as_ C—H, CH_)_, 1380(*m*) (δ_s_ C—H, CH_3_), 1095(*s*) (ν C—O, alcohol), 1010(*s*) 910(*s*) (δ C—H, olefin); Anal. Calcd. for C_11_H_18_O_3_: C, 66.6; H, 9.2; Found: C, 66.6; H, 9.4; [α]_D_^20^ +16.0 (c 0.6, CHCl_3_); C_11_H_18_O_3_, *M* = 198.26 g/mol.

### Crystal data

**Table:**

C_11_H_18_O_3_	*F*(000) = 216
*M**_r_* = 198.25	*D*_x_ = 1.190 Mg m^−^^3^
Monoclinic, *P*2_1_	Mo *K*α radiation, λ = 0.71073 Å
Hall symbol: P 2yb	Cell parameters from 1159 reflections
*a* = 7.7265 (10) Å	θ = 2.6–29.1°
*b* = 6.4798 (8) Å	µ = 0.09 mm^−^^1^
*c* = 11.0598 (16) Å	*T* = 173 K
β = 92.563 (14)°	Plate, colourless
*V* = 553.17 (13) Å^3^	0.50 × 0.18 × 0.04 mm
*Z* = 2	

### Data collection

**Table:**

Oxford Diffraction Xcalibur S CCD diffractometer	737 reflections with *I* > 2σ(*I*)
Radiation source: Enhance (Mo) X-ray Source	*R*_int_ = 0.040
graphite	θ_max_ = 25.5°, θ_min_ = 2.6°
Detector resolution: 16.0560 pixels mm^-1^	*h* = −10→10
ω scans	*k* = −7→8
3255 measured reflections	*l* = −14→14
1129 independent reflections	

### Refinement

**Table:**

Refinement on *F*^2^	Primary atom site location: structure-invariant direct methods
Least-squares matrix: full	Secondary atom site location: difference Fourier map
*R*[*F*^2^ > 2σ(*F*^2^)] = 0.035	Hydrogen site location: inferred from neighbouring sites
*wR*(*F*^2^) = 0.048	H-atom parameters constrained
*S* = 0.99	*w* = 1/[σ^2^(*F*_o_^2^) + (0.011*P*)^2^] where *P* = (*F*_o_^2^ + 2*F*_c_^2^)/3
1129 reflections	(Δ/σ)_max_ < 0.001
131 parameters	Δρ_max_ = 0.14 e Å^−^^3^
1 restraint	Δρ_min_ = −0.15 e Å^−^^3^

### Special details

**Table:**

Geometry. All e.s.d.'s (except the e.s.d. in the dihedral angle between two l.s. planes) are estimated using the full covariance matrix. The cell e.s.d.'s are taken into account individually in the estimation of e.s.d.'s in distances, angles and torsion angles; correlations between e.s.d.'s in cell parameters are only used when they are defined by crystal symmetry. An approximate (isotropic) treatment of cell e.s.d.'s is used for estimating e.s.d.'s involving l.s. planes.
Refinement. Refinement of *F*^2^ against ALL reflections. The weighted *R*-factor *wR* and goodness of fit *S* are based on *F*^2^, conventional *R*-factors *R* are based on *F*, with *F* set to zero for negative *F*^2^. The threshold expression of *F*^2^ > σ(*F*^2^) is used only for calculating *R*-factors(gt) *etc*. and is not relevant to the choice of reflections for refinement. *R*-factors based on *F*^2^ are statistically about twice as large as those based on *F*, and *R*- factors based on ALL data will be even larger.

### Fractional atomic coordinates and isotropic or equivalent isotropic displacement parameters (Å^2^)

**Table:**

	*x*	*y*	*z*	*U*_iso_*/*U*_eq_	
O1	0.2173 (2)	0.1438 (2)	0.07628 (15)	0.0251 (5)	
O2	0.2731 (2)	0.0175 (3)	−0.10413 (17)	0.0328 (6)	
O3	0.1599 (2)	0.6838 (3)	0.05289 (17)	0.0370 (6)	
H3	0.2032	0.7730	0.0082	0.056*	
C1	0.2453 (3)	0.1658 (5)	−0.0428 (2)	0.0267 (8)	
C2	0.2329 (4)	0.3900 (4)	−0.0771 (2)	0.0251 (8)	
H2	0.1128	0.4168	−0.1109	0.030*	
C3	0.2533 (3)	0.4969 (4)	0.0446 (2)	0.0275 (8)	
H3A	0.3790	0.5230	0.0645	0.033*	
C4	0.1847 (4)	0.3431 (4)	0.1331 (3)	0.0251 (7)	
H4	0.0569	0.3630	0.1382	0.030*	
C5	0.2674 (4)	0.3375 (4)	0.2598 (2)	0.0293 (8)	
H5	0.3954	0.3280	0.2512	0.035*	
C6	0.2135 (4)	0.1458 (4)	0.3322 (2)	0.0314 (8)	
H6	0.2292	0.0238	0.2784	0.038*	
C7	0.3345 (4)	0.1158 (5)	0.4405 (3)	0.0409 (9)	
H7	0.4546	0.1147	0.4255	0.049*	
C8	0.2950 (4)	0.0912 (5)	0.5523 (2)	0.0487 (10)	
H8A	0.1770	0.0910	0.5730	0.058*	
H8B	0.3841	0.0735	0.6134	0.058*	
C9	0.3607 (3)	0.4531 (4)	−0.1730 (2)	0.0350 (9)	
H9A	0.4789	0.4182	−0.1443	0.053*	
H9B	0.3523	0.6022	−0.1872	0.053*	
H9C	0.3323	0.3793	−0.2487	0.053*	
C10	0.2329 (4)	0.5418 (4)	0.3243 (2)	0.0401 (9)	
H10A	0.2922	0.6541	0.2836	0.060*	
H10B	0.2765	0.5329	0.4087	0.060*	
H10C	0.1080	0.5689	0.3218	0.060*	
C11	0.0231 (3)	0.1493 (5)	0.3622 (2)	0.0434 (9)	
H11A	−0.0082	0.0167	0.3979	0.065*	
H11B	−0.0484	0.1728	0.2881	0.065*	
H11C	0.0033	0.2605	0.4201	0.065*	

### Atomic displacement parameters (Å^2^)

**Table:**

	*U*^11^	*U*^22^	*U*^33^	*U*^12^	*U*^13^	*U*^23^
O1	0.0324 (13)	0.0134 (13)	0.0295 (11)	−0.0005 (11)	0.0026 (10)	−0.0004 (11)
O2	0.0385 (14)	0.0195 (12)	0.0403 (14)	−0.0003 (11)	0.0013 (11)	−0.0058 (12)
O3	0.0427 (14)	0.0116 (11)	0.0573 (15)	0.0053 (11)	0.0087 (11)	0.0052 (12)
C1	0.0145 (16)	0.0214 (18)	0.044 (2)	−0.0032 (17)	−0.0048 (15)	0.003 (2)
C2	0.0219 (17)	0.0155 (18)	0.038 (2)	−0.0004 (14)	0.0015 (15)	0.0037 (15)
C3	0.0222 (18)	0.0125 (16)	0.048 (2)	0.0004 (16)	0.0007 (16)	0.0048 (16)
C4	0.0229 (17)	0.0143 (16)	0.0380 (19)	0.0038 (15)	0.0020 (15)	−0.0005 (16)
C5	0.0261 (17)	0.0224 (19)	0.039 (2)	0.0040 (16)	−0.0001 (16)	−0.0068 (17)
C6	0.048 (2)	0.0133 (19)	0.0330 (18)	0.0029 (17)	0.0011 (16)	−0.0060 (17)
C7	0.049 (2)	0.038 (2)	0.0359 (17)	0.0078 (19)	0.0001 (17)	0.0032 (18)
C8	0.054 (3)	0.052 (2)	0.039 (2)	−0.003 (2)	−0.005 (2)	0.0019 (19)
C9	0.0337 (19)	0.0271 (19)	0.045 (2)	−0.0014 (17)	0.0090 (16)	0.0070 (17)
C10	0.046 (2)	0.025 (2)	0.049 (2)	−0.0045 (17)	0.0060 (18)	−0.0113 (17)
C11	0.049 (2)	0.037 (2)	0.0442 (19)	−0.010 (2)	0.0005 (17)	0.007 (2)

### Geometric parameters (Å, °)

**Table:**

O1—C1	1.352 (3)	C6—C7	1.499 (3)
O1—C4	1.463 (3)	C6—C11	1.523 (3)
O2—C1	1.201 (3)	C6—H6	1.0000
O3—C3	1.415 (3)	C7—C8	1.296 (3)
O3—H3	0.8400	C7—H7	0.9500
C1—C2	1.504 (4)	C8—H8A	0.9500
C2—C3	1.516 (3)	C8—H8B	0.9500
C2—C9	1.536 (3)	C9—H9A	0.9800
C2—H2	1.0000	C9—H9B	0.9800
C3—C4	1.510 (3)	C9—H9C	0.9800
C3—H3A	1.0000	C10—H10A	0.9800
C4—C5	1.515 (3)	C10—H10B	0.9800
C4—H4	1.0000	C10—H10C	0.9800
C5—C10	1.533 (3)	C11—H11A	0.9800
C5—C6	1.544 (4)	C11—H11B	0.9800
C5—H5	1.0000	C11—H11C	0.9800
			
C1—O1—C4	111.3 (2)	C7—C6—C5	110.2 (2)
C3—O3—H3	109.5	C11—C6—C5	112.7 (2)
O2—C1—O1	120.4 (3)	C7—C6—H6	106.6
O2—C1—C2	129.9 (2)	C11—C6—H6	106.6
O1—C1—C2	109.7 (3)	C5—C6—H6	106.6
C1—C2—C3	102.4 (2)	C8—C7—C6	127.8 (3)
C1—C2—C9	113.4 (2)	C8—C7—H7	116.1
C3—C2—C9	116.5 (2)	C6—C7—H7	116.1
C1—C2—H2	108.1	C7—C8—H8A	120.0
C3—C2—H2	108.1	C7—C8—H8B	120.0
C9—C2—H2	108.1	H8A—C8—H8B	120.0
O3—C3—C4	109.1 (2)	C2—C9—H9A	109.5
O3—C3—C2	114.6 (2)	C2—C9—H9B	109.5
C4—C3—C2	104.4 (2)	H9A—C9—H9B	109.5
O3—C3—H3A	109.5	C2—C9—H9C	109.5
C4—C3—H3A	109.5	H9A—C9—H9C	109.5
C2—C3—H3A	109.5	H9B—C9—H9C	109.5
O1—C4—C3	103.4 (2)	C5—C10—H10A	109.5
O1—C4—C5	107.6 (2)	C5—C10—H10B	109.5
C3—C4—C5	118.0 (2)	H10A—C10—H10B	109.5
O1—C4—H4	109.1	C5—C10—H10C	109.5
C3—C4—H4	109.1	H10A—C10—H10C	109.5
C5—C4—H4	109.1	H10B—C10—H10C	109.5
C4—C5—C10	109.6 (2)	C6—C11—H11A	109.5
C4—C5—C6	112.7 (2)	C6—C11—H11B	109.5
C10—C5—C6	113.4 (2)	H11A—C11—H11B	109.5
C4—C5—H5	106.9	C6—C11—H11C	109.5
C10—C5—H5	106.9	H11A—C11—H11C	109.5
C6—C5—H5	106.9	H11B—C11—H11C	109.5
C7—C6—C11	113.6 (2)		
			
C4—O1—C1—O2	−179.5 (2)	C2—C3—C4—O1	28.3 (3)
C4—O1—C1—C2	−0.1 (3)	O3—C3—C4—C5	−90.1 (3)
O2—C1—C2—C3	−162.7 (3)	C2—C3—C4—C5	147.0 (2)
O1—C1—C2—C3	18.0 (3)	O1—C4—C5—C10	−178.7 (2)
O2—C1—C2—C9	−36.3 (4)	C3—C4—C5—C10	64.9 (3)
O1—C1—C2—C9	144.4 (2)	O1—C4—C5—C6	−51.4 (3)
C1—C2—C3—O3	−147.4 (2)	C3—C4—C5—C6	−167.8 (2)
C9—C2—C3—O3	88.3 (3)	C4—C5—C6—C7	163.9 (2)
C1—C2—C3—C4	−28.1 (3)	C10—C5—C6—C7	−70.9 (3)
C9—C2—C3—C4	−152.4 (2)	C4—C5—C6—C11	−68.0 (3)
C1—O1—C4—C3	−18.0 (3)	C10—C5—C6—C11	57.3 (3)
C1—O1—C4—C5	−143.6 (2)	C11—C6—C7—C8	0.8 (5)
O3—C3—C4—O1	151.2 (2)	C5—C6—C7—C8	128.5 (4)

### Hydrogen-bond geometry (Å, °)

**Table:**

*D*—H···*A*	*D*—H	H···*A*	*D*···*A*	*D*—H···*A*
O3—H3···O1^i^	0.84	2.52	3.023 (2)	120
O3—H3···O2^i^	0.84	2.10	2.931 (3)	171

Symmetry codes: (i) *x*, *y*+1, *z*.
